# Depletion of regulatory T cells leads to an exacerbation of delayed-type hypersensitivity arthritis in C57BL/6 mice that can be counteracted by IL-17 blockade

**DOI:** 10.1242/dmm.022905

**Published:** 2016-04-01

**Authors:** Sara Marie Atkinson, Ute Hoffmann, Alf Hamann, Emil Bach, Niels Banhos Danneskiold-Samsøe, Karsten Kristiansen, Kyle Serikawa, Brian Fox, Kim Kruse, Claus Haase, Søren Skov, Anneline Nansen

**Affiliations:** 1 Department of Diabetes Complications Research, Global Research, Novo Nordisk A/S, Maaloev 2760, Denmark; 2 Department of Veterinary Disease Biology, University of Copenhagen, Frederiksberg 1870, Denmark; 3 Experimentelle Rheumatologie, Deutsches Rheuma-Forschungszentrum and Charité-Universitätsmedizin Berlin, Berlin 10117, Germany; 4 Laboratory of Genomics and Molecular Biomedicine, Department of Biology, University of Copenhagen, Copenhagen 2100, Denmark; 5 Benaroya Research Institute, Seattle, WA 98101, USA; 6 Immunexpress, Seattle, WA 98109, USA; 7 Department of Pharmacology, Zealand Pharma, Glostrup 2600, Denmark

**Keywords:** Rheumatoid arthritis, Regulatory T cells, IL-17, Neutrophils, C57BL/6, ACPA, Microbiota

## Abstract

Rodent models of arthritis have been extensively used in the elucidation of rheumatoid arthritis (RA) pathogenesis and are instrumental in the development of therapeutic strategies. Here we utilise delayed-type hypersensitivity arthritis (DTHA), a model in C57BL/6 mice affecting one paw with synchronised onset, 100% penetrance and low variation. We investigate the role of regulatory T cells (T_regs_) in DTHA through selective depletion of T_regs_ and the role of IL-17 in connection with T_reg_ depletion. Given the relevance of T_regs_ in RA, and the possibility of developing T_reg_-directed therapies, this approach could be relevant for advancing the understanding of T_regs_ in inflammatory arthritis. Selective depletion of T_regs_ was achieved using a *Foxp3-DTR-eGFP* mouse, which expresses the diphtheria toxin receptor (DTR) and enhanced green fluorescent protein (eGFP) under control of the *Foxp3* gene. Anti-IL-17 monoclonal antibody (mAb) was used for IL-17 blockade. Numbers and activation of T_regs_ increased in the paw and its draining lymph node in DTHA, and depletion of T_regs_ resulted in exacerbation of disease as shown by increased paw swelling, increased infiltration of inflammatory cells, increased bone remodelling and increased production of inflammatory mediators, as well as increased production of anti-citrullinated protein antibodies. Anti-IL-17 mAb treatment demonstrated that IL-17 is important for disease severity in both the presence and absence of T_regs_, and that IL-17 blockade is able to rescue mice from the exacerbated disease caused by T_reg_ depletion and caused a reduction in RANKL, IL-6 and the number of neutrophils. We show that T_regs_ are important for the containment of inflammation and bone remodelling in DTHA. To our knowledge, this is the first study using the *Foxp3-DTR-eGFP* mouse on a C57BL/6 background for T_reg_ depletion in an arthritis model, and we here demonstrate the usefulness of the approach to study the role of T_regs_ and IL-17 in arthritis.

## Abstract (precis)

**Summary:** This paper increases the understanding of disease drivers and regulators in delayed-type hypersensitivity arthritis, a robust and reproducible arthritis model in C57BL/6 mice.

## INTRODUCTION

Rodent models of arthritis have been extensively used in the process of elucidating the pathogenesis of rheumatoid arthritis (RA), a chronic inflammatory disease characterised by chronic joint inflammation and bone erosion, and they are instrumental in the development of new therapeutic strategies (Asquith et al., 2009). Delayed-type hypersensitivity arthritis (DTHA) is an arthritis model in C57BL/6 (B6) mice that is characterised by synchronised onset, 100% penetrance and low variation. DTHA is induced by modifying a protein-antigen-induced delayed-type hypersensitivity response in the paw by administration of an anti-type-II-collagen antibody cocktail (anti-CII) between immunisation and challenge. The result is severe inflammatory arthritis affecting one, pre-defined, hind paw (Atkinson et al., 2012). Notably, the anti-CII dose required for DTHA is five- to eight-times lower than required for induction of collagen-antibody-induced arthritis (CAIA) in B6 mice (Hutamekalin et al., 2009).

Owing to their well-established role in controlling inflammation in many animal models, we hypothesise that regulatory T cells (T_regs_) play a role in the resolution of inflammation in DTHA. It has been shown in mouse models of arthritis that deficiency or depletion of T_regs_ exacerbates disease, and increasing numbers of T_regs_ can reduce disease activity (Gizinski and Fox, 2014; Kelchtermans et al., 2009; Miyara et al., 2014; Noack and Miossec, 2014; Zaiss et al., 2010)*.* The development of a more sustained disease phenotype in the absence of T_regs_ would let us study disease drivers unchecked by this immunoregulatory cell subset and to identify which disease driver mechanisms are suppressed by T_regs_ *in vivo* in experimental arthritis. The purpose of the present study was therefore to investigate the mechanisms of self-limiting disease in DTHA through selective depletion of T_regs_ after disease induction. T_reg_ depletion studies in experimental arthritis models are scant and have mainly used the anti-CD25 approach. Anti-CD25 treatment in collagen-induced arthritis (CIA) accelerates disease (Kelchtermans et al., 2005; Morgan et al., 2003). Administration of anti-CD25 prior to induction exacerbates glucose-6-phospate isomerase (G6PI)-induced arthritis (Frey et al., 2010) and antigen-induced arthritis (AIA) (Frey et al., 2005). However, using anti-CD25 antibodies for depletion of T_regs_ also targets effector T cells (T_eff_). The *Foxp3-DTR-eGFP* mouse allows selective depletion of T_regs_ without affecting T_eff_. This mouse expresses a fusion of a diphtheria toxin receptor (DTR) and enhanced green fluorescent protein (eGFP) under the control of the forkhead box protein 3 (*Foxp3*) gene locus, which means that T_regs_ can be selectively depleted by administration of diphtheria toxin (DT) (Feuerer et al., 2009; Lahl et al., 2007). T_reg_ depletion in *Foxp3-DTR-eGFP* mice on a DBA/1 background exacerbated G6PI-induced arthritis (Irmler et al., 2014). However, to our knowledge, this is the first study to address, in-depth, the use of the *Foxp3-DTRe-GFP* mouse for T_reg_ depletion in experimental arthritis and the first to use the *Foxp3-DTR-eGFP* mouse on a B6 background in experimental arthritis.

RA has recently been associated with changes in the gut microbiota (Scher et al., 2013; Zhang et al., 2015). In spontaneous mouse models of autoimmune arthritis, joint inflammation is attenuated under germ-free conditions, but colonisation of the gut with commensal microbes is sufficient to elicit joint inflammation comparable to that observed in conventional mice (Abdollahi-Roodsaz et al., 2008; Wu et al., 2010). In these models, the colonisation of the gut resulted in a perturbed T_reg_/T_eff_ balance, which was associated with disease onset and progression. The interplay between T_regs_ and the gut microbiota modulates T_reg_ abundance and function and might thereby also affect onset and progression of arthritis. Therefore, we also analysed the fecal microbiota following DTHA induction alone and in conjunction with T_reg_ depletion.

In the present study, we found that depleting T_regs_ after onset of DTHA led to an exacerbation of arthritis. Inflammatory cell infiltration, osteoclast activation and bone erosion were increased in T_reg_-depleted mice. T_reg_ depletion also increased levels of anti-mutated-citrullinated-vimentin (MCV) antibodies, indicating an increase in autoimmunity associated with increased protein citrullination. Production of a wide range of cytokines and chemokines was increased, including IL-17, a cytokine involved in the pathogenesis of both RA and murine experimental arthritis. We show that treating T_reg_-depleted mice with anti-IL-17 monoclonal antibody (mAb) rescues them from exacerbation of disease through a reduction of circulating neutrophils and a reduction in IL-6 and receptor-activator of nuclear factor κB ligand (RANKL).

## RESULTS

### A highly activated and proliferating subset of T_regs_ is found in the lymph node draining the arthritic paw early after disease onset

**Fig. 1.**
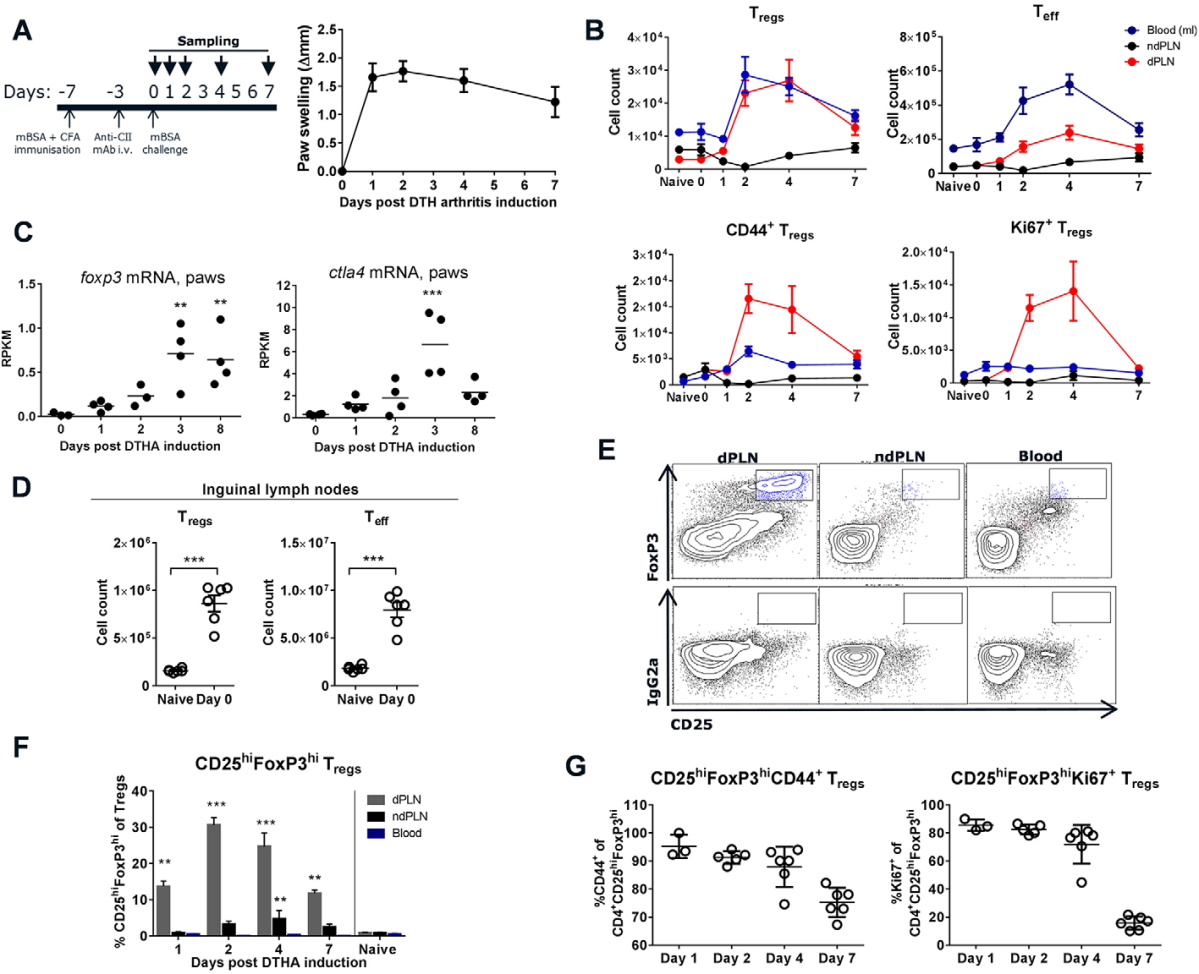
**Dynamics and phenotypic characterisation of regulatory T cells (T_regs_) during delayed-type hypersensitivity arthritis (DTHA).** (A) DTHA was induced by immunising mice with methylated bovine serum albumin (mBSA) emulsified in complete Freund's adjuvant (CFA) on day −7, followed by an intravenous (i.v.) dose of an anti-type-II-collagen antibody cocktail (anti-CII) on day −3. On day 0 the mice were challenged with mBSA in the right hind paw and with vehicle in the left hind paw. Δpaw swelling on day *x* was calculated by subtracting the baseline measurement made on day 0 prior to challenge from the measurement made on day *x*. *n*=5-35; mean±s.d. shown. (B) T_regs_ were defined as live CD45^+^TCRβ^+^CD4^+^CD25^+^FoxP3^+^ cells, and effector T cells (T_eff_) by inverting the CD25^+^FoxP3^+^ gate within the CD45^+^TCRβ^+^CD4^+^ gate. T_regs_ were further gated on the activation marker CD44 and the proliferation marker Ki67. Total numbers of T_regs_, T_eff_, CD44^+^ T_regs_ and Ki67^+^ T_regs_ are shown in blood (cells/ml), the popliteal lymph node draining the arthritic paw (dPLN) and the popliteal lymph node draining the control paw (ndPLN). Mean±s.e.m. is shown, *n*=5. (C) *Foxp3* and *Ctla4* mRNA in arthritic paws measured by mRNA deep sequencing. RPKM, reads per kilobase of transcript per million mapped reads. Mean shown, *n*=4. **P≤0.01, ***P≤0.001, difference to levels on day 0, one-way ANOVA. (D) Total cell counts in the inguinal lymph nodes. T_regs_ were defined as live CD45^+^TCRβ^+^CD4^+^CD25^+^FoxP3^+^ cells and T_eff_ by inverting the CD25^+^FoxP3^+^ gate within the CD45^+^TCRβ^+^CD4^+^ gate, *n*=6, mean±s.e.m. shown. Student's *t*-test, ***P≤0.001. (E) Representative flow cytometry plots from day 2 post-DTHA induction showing CD25^hi^FoxP3^hi^ T_reg_ populations (box) in dPLN, ndPLN and blood. Cells were defined as live CD45^+^TCRβ^+^CD4^+^ before gating on CD25 and FoxP3. The top panel shows FoxP3 staining and the bottom panel staining with IgG2a isotype control antibody. (F) Percentage of CD25^hi^FoxP3^hi^ T_regs_ in dPLN, ndPLN and blood after induction of DTHA and in naïve mice. Mean±s.e.m. shown, *n*=3-6. Difference to percentages in naïve mice analysed using one-way ANOVA, **P≤0.01, ***P≤0.001. (G) Percentage of CD25^hi^FoxP3^hi^ T_regs_ in dPLN expressing CD44 (left) and Ki67 (right); mean±s.d. shown, *n*=3-6. All experiments presented in this figure were carried out once. Full flow cytometry gating strategy is shown in Fig. S1A.

We investigated the dynamics of T_regs_ in DTHA by analysing the popliteal lymph node draining the arthritic paw (dPLN), the popliteal lymph node draining the control paw (ndPLN), paw infiltrate and blood at different time points during disease by flow cytometry (Fig. 1A). T_regs_ were defined as live CD45^+^TCRβ^+^CD4^+^CD25^+^FoxP3^+^ cells and T_eff_ cells by inverting the CD25^+^FoxP3^+^ gate within the CD45^+^TCRβ^+^CD4^+^ gate (full gating strategy shown in Fig. S1A). T_regs_ were most abundant in the dPLN on day 2 and 4 after DTHA induction and were still elevated compared to day 1 and naïve mice on day 7. The increase in T_reg_ number in the dPLN was also reflected in blood (Fig. 1B). A dramatic increase in activated (CD44^+^) and proliferating (Ki67^+^) T_regs_ was seen in the dPLN, but not in the ndPLN or in blood (Fig. 1B, bottom graphs). Analysis of *Foxp3* mRNA showed a significantly higher *Foxp3* expression in paws on day 3 and 8 compared to before arthritis induction (Fig. 1C). The same was seen with *Ctla4* mRNA, which codes for the surface marker cytotoxic T-lymphocyte-associated protein 4 (CTLA4) found on T_regs_ and memory T cells, but to a greater extent on T_regs_ (Jago et al., 2004) (Fig. 1C). No increase in T_reg_ numbers in blood or popliteal lymph nodes was observed during the immunisation phase of DTHA (from naïve to day 0), so we also analysed the inguinal lymph nodes (ILNs), which drain the immunisation sites, in naïve mice and immediately prior to methylated bovine serum albumin (mBSA) challenge. In these lymph nodes a dramatic increase in the frequency of both T_reg_ and T_eff_ cells was observed (Fig. 1D), but this increase was not reflected in the number of circulating T_regs_ or T_eff_ on day 0 (Fig. 1B). Interestingly, a subpopulation of CD25^hi^FoxP3^hi^ T_regs_ was found in the dPLN but not in the ndPLN or blood (Fig. 1E,F). These CD25^hi^FoxP3^hi^ T_regs_ were found to possess an activated phenotype and were actively proliferating already from day 1 post-DTHA induction (Fig. 1G). We also analysed cells isolated from the arthritic paw on day 2 and 4 after DTHA induction and found the fraction of T_regs_ of total T cells to increase from 12.17±1.09% to 14.70±0.63% from day 2 to 4 (mean±s.e.m., *P*=0.079, Student's *t*-test). Analysis of immune cells isolated from non-inflamed paws is not feasible owing to the very low number of cells that it is possible to harvest. We also found subpopulations of CD25^hi^FoxP3^hi^ and Ki67^+^ T_regs_ in the paw. Among paw infiltrating T cells on day 2 and day 4, we found 33.72±4.16% and 23.04±1.55% of T_regs_ to be CD25^hi^FoxP3^hi^ (mean±s.e.m., *P*=0.044, Student's *t*-test) and 82.16±1.3% and 86.35±1.2% of T_regs_ to be Ki67^+^ (mean±s.e.m., *P*=0.034, Student's *t*-test), respectively. In summary, we observed increased numbers of activated and proliferating T_regs_ in the dPLN and paw early after mBSA challenge in DTHA.

### Depletion of T_regs_ after arthritis induction exacerbates inflammation and leads to a rapid increase in arthritic disease activity

**Fig. 2.**
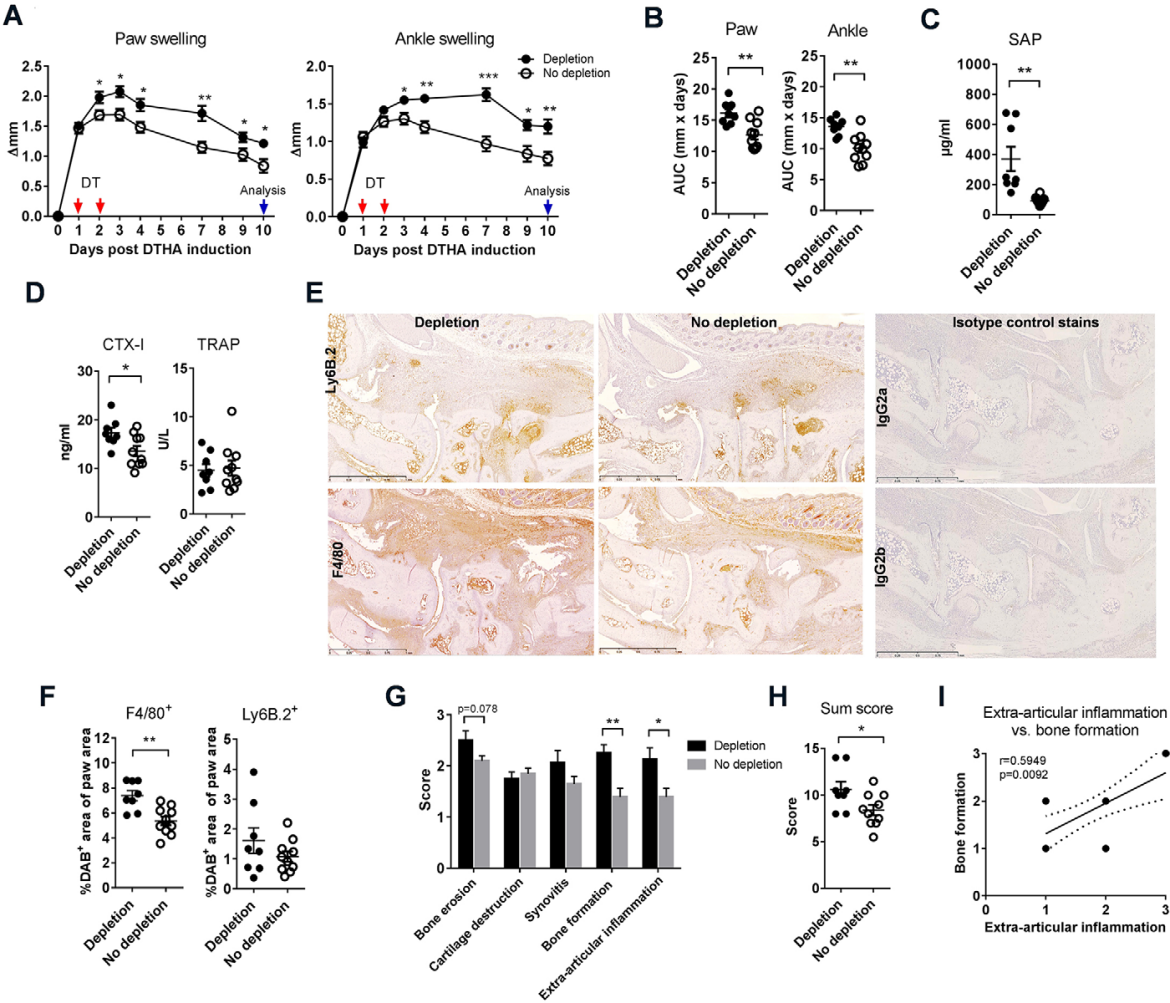
**Depletion of T_regs_ leads to increased disease activity in DTHA.** T_regs_ were depleted from mice carrying the *Foxp3-DTR-eGFP* transgene (FoxP3-DTR^+^) by administration of diphtheria toxin (DT) 24 and 48 h after DTHA induction. Non-transgene littermates (FoxP3-DTR^−^) were used as controls and also given DT. Paw and ankle swelling shown over the duration of the study (A) and as area under curve (AUC) (B), *n*=8-10, mean±s.e.m. shown. Student's *t*-test, *P≤0.05; **P≤0.01; ***P≤0.001. (C) Serum levels of serum amyloid P component (SAP) in serum measured 10 days after DTHA induction by ELISA, *n*=8-10, mean±s.e.m. shown. Student's *t*-test, **P≤0.01. (D) Serum levels of C-terminal telopeptide of type I collagen (CTX-I) and tartrate-resistant acid phosphatase (TRAP) measured 10 days after DTHA induction by ELISA, *n*=8-10, mean±s.e.m. shown. Student's *t*-test, *P≤0.05. (E) Representative images of immunohistochemistry (IHC) stainings for the macrophage marker F4/80 or the neutrophil marker Ly6B.2. Positively stained cells appear brown. Scale bars: 1 mm. (F) Macrophage and neutrophil infiltration quantified by analysing paw sections stained by immunohistochemistry for the macrophage marker F4/80 or the neutrophil marker Ly6B.2 using digitalised image analysis software. Target-cell infiltration was defined as percentage of 3-3′-diamino-benzidine-tetrahydrochloride-positive (DAB^+^) area of total paw area. *n*=8-10, mean±s.e.m. shown. Student's *t*-test, **P≤0.01. (G) Semi-quantitative histopathological scoring of arthritic and inflammatory parameters on day 10 using a scale of 0-3 (see Materials and Methods for details), *n*=8-10, mean±s.e.m. shown. Student's *t*-test with Welch's correction, *P≤0.05; **P≤0.01. (H) Sum of the individual scores in G. Maximum possible score is 15. *n*=8-10, mean±s.e.m. shown. Student's *t*-test with Welch's correction, *P≤0.05. (I) Correlation between the histopathological scores for extra-articular inflammation and bone formation in all mice. Slope±95% CI shown, *n*=18 (some points are on top of each other). Spearman *r*=0.5949, *P*=0.0092. Results presented in this figure are representative of two independent experiments.

In order to assess the importance of T_regs_ in regulating DTHA severity, we selectively depleted T_regs_ by administration of DT at 24 and 48 h after arthritis induction to *Foxp3-DTR-eGFP* mice (FoxP3-DTR^+^) and littermate controls (FoxP3-DTR^−^). This resulted in depletion of all eGFP^+^ cells in FoxP3-DTR^+^ mice as confirmed by flow cytometry (Fig. S2). However, eGFP^+^ cells eventually repopulate the T_reg_ niche after transient DT treatment, as shown in Fig. S2C. Depletion of T_regs_ rapidly led to increased paw and ankle swelling, which persisted up to at least day 10 (Fig. 2A,B). In T_reg_-depleted mice, levels of the acute-phase protein serum amyloid P component (SAP) were elevated in serum on day 10 compared to control mice (Fig. 2C), as was C-terminal telopeptide of type I collagen (CTX-I; Fig. 2D), whereas tartrate-resistant acid phosphatase (TRAP) serum levels were not increased (Fig. 2D). Histopathological analysis on day 10 showed increased infiltration of macrophages into the T_reg_-depleted paws (Fig. 2E,F). Histological scoring revealed a tendency towards increased bone erosion in T_reg_-depleted mice, whereas there was no difference in cartilage degradation and synovitis (Fig. 2G). However, inflammation and new bone formation at extra-articular sites were increased in T_reg_-depleted mice, and so was the histopathological sum score (Fig. 2H). Interestingly, we observed a correlation (*r*=0.5949, *P*=0.0092, *n*=18) between extra-articular inflammation and new bone formation (Fig. 2I), indicating that new bone formation in DTHA is an inflammation-associated process.

### Depletion of T_regs_ leads to an increase in biomarkers of arthritis and inflammatory cells in the affected paw and in circulation

**Fig. 3.**
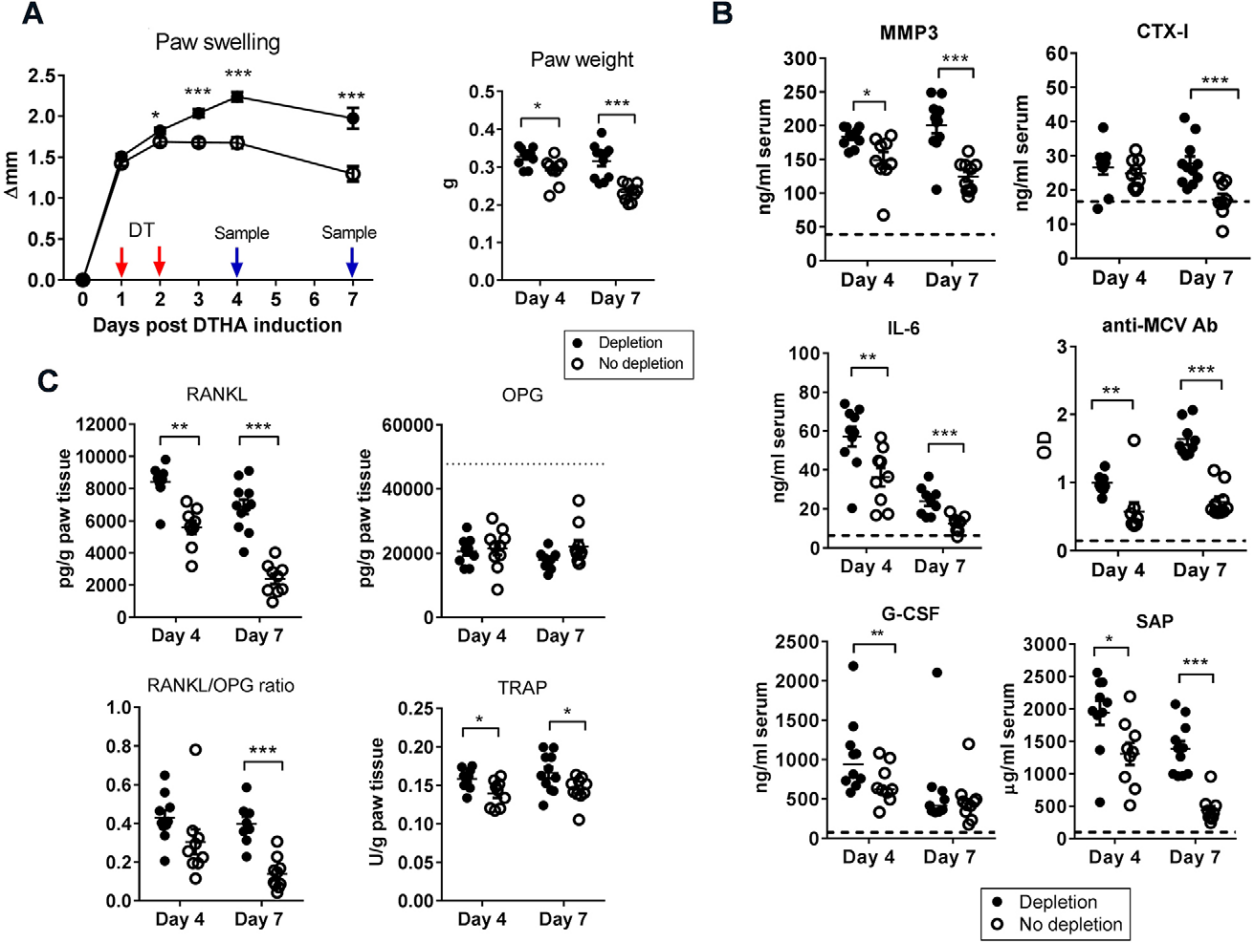
**Depletion of T_regs_ leads to rapidly increased inflammatory and arthritic activity both locally and systemically.** (A) T_regs_ were depleted from mice carrying the *Foxp3-DTR-eGFP* transgene (FoxP3-DTR^+^) by administration of diphtheria toxin (DT) 24 and 48 h after DTHA induction. Non-transgene littermates (FoxP3-DTR^−^) were used as controls. Paw swelling shown over the duration of the study (left) and as paw weight (right). Mean±s.e.m. shown, *n*=10. Student's *t*-test, *P≤0.05; ***P≤0.001. (B) Serum levels of matrix metalloproteinase 3 (MMP3), C-terminal telopeptide of type I collagen (CTX-I), IL-6, antibodies to mutated citrullinated vimentin (anti-MCV), granulocyte colony stimulating factor (G-CSF) and serum amyloid P component (SAP) were measured by ELISA in serum on day 4 and 7 post-DTHA induction. Mean±s.e.m. shown, *n*=10. Student's *t*-test, *P≤0.05; **P≤0.01; ***P≤0.001. (C) Levels of receptor-activator of nuclear factor kappa B ligand (RANKL), osteoprotegerin (OPG) and tartrate-resistant acid phosphatase (TRAP) were measured by ELISA in whole paw homogenate supernatants on day 4 and 7 after DTHA induction. Mean±s.e.m. shown, *n*=10. Student's *t*-test, *P≤0.05; **P≤0.01; ***P≤0.001. Dotted line represents mean OPG level in *n*=2 naïve mice. Results presented in this figure are representative of two independent experiments.

**Fig. 4.**
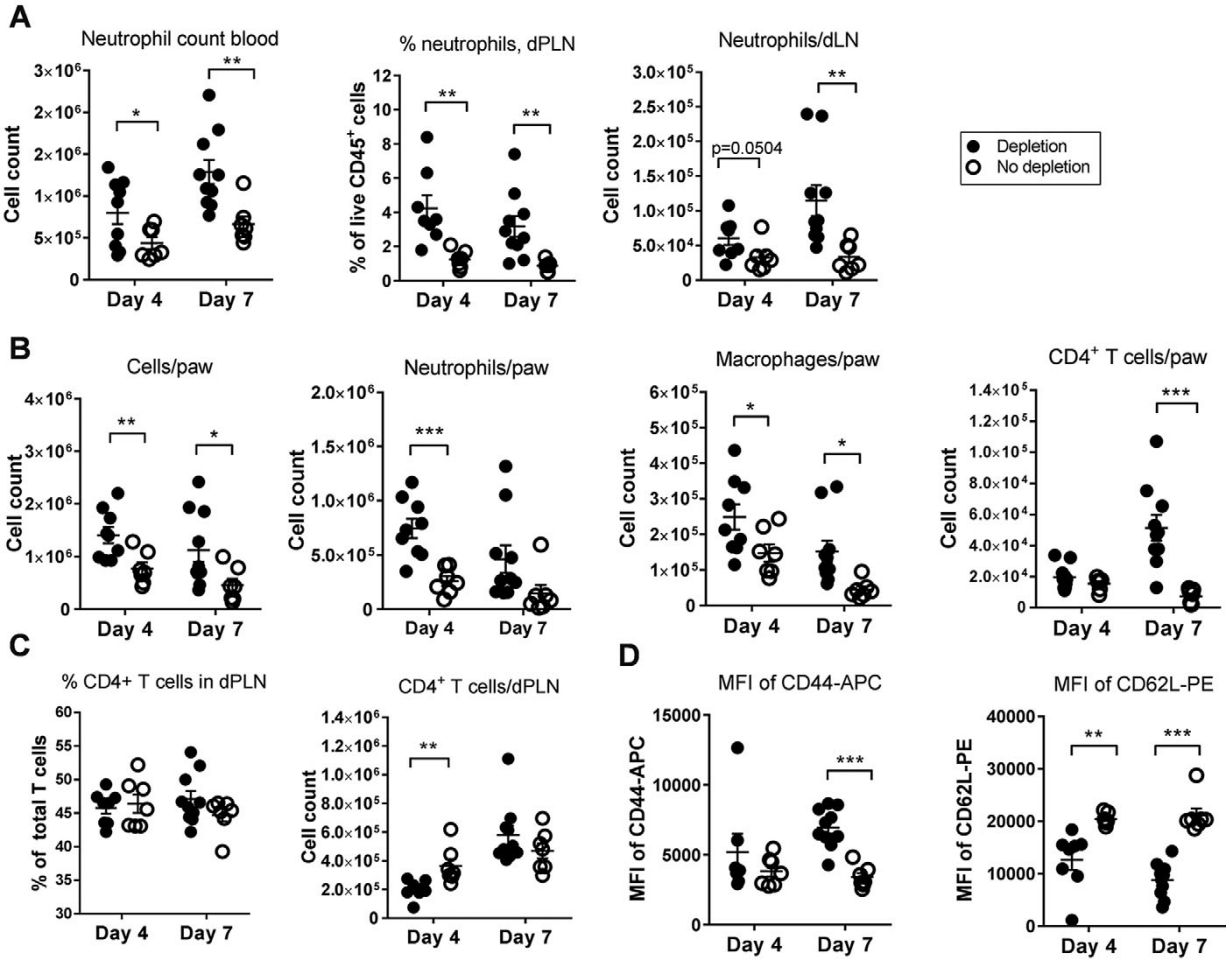
**T_reg_ depletion in DTHA leads to increased neutrophil recruitment and CD4^+^ T-cell activity.** (A) Counts and percentages of neutrophils were analysed in blood and the popliteal lymph node draining the arthritic paw (dPLN) on day 4 and 7 after DTHA induction by flow cytometry. Neutrophils were defined as live CD45^+^CD11b^+^Ly6G^+^. Mean±s.e.m. shown, *n*=10. Student's *t*-test, *P≤0.05; **P≤0.01. (B) Cell counts of total live cells, CD4^+^ T cells, macrophages and neutrophils in the arthritic paw analysed on day 4 and 7 after DTHA induction by flow cytometry. CD4^+^ T cells and neutrophils defined as before, macrophages defined as live CD45^+^CD11b^+^F4/80^+^ cells. Mean±s.e.m. shown, *n*=10. Student's *t*-test, *P≤0.05; **P≤0.01; ***P≤0.001. (C) Counts and percentages of CD4^+^ T cells analysed in the dPLN on day 4 and 7 after DTHA induction by flow cytometry. CD4^+^ T cells were defined as live CD45^+^TCRβ^+^CD4^+^ cells. Mean±s.e.m. shown, *n*=10. Student's *t*-test, **P≤0.01. (D) Median fluorescence intensity (MFI) of CD44-APC (left) and CD62L-PE (right) in the CD4^+^ T-cell gate. Mean±s.e.m. shown, *n*=10. Student's *t*-test, **P≤0.01; ***P≤0.001. Results presented in this figure are representative of two independent experiments. Full flow cytometry gating strategy is shown in Fig. S1B,C.

Next, we investigated the mechanisms of the increased arthritis response following T_reg_ depletion by sampling on day 4 and 7 post-arthritis induction. We found increased paw swelling in T_reg_-depleted mice (Fig. 3A), and increased levels of SAP, IL-6 and matrix metalloproteinase 3 (MMP3) on both day 4 and 7 in T_reg_-depleted mice (Fig. 3B). Levels of CTX-I were increased on day 7 but not day 4. Neutrophil chemoattractant granulocyte colony stimulating factor (G-CSF) was increased on day 4, but not day 7 (Fig. 3B). Interestingly, we detected production of anti-MCV in DTHA, and, following T_reg_ depletion, anti-MCV antibody levels were increased in serum (Fig. 3B). In order to investigate whether T_regs_ play a role in regulating bone resorption in DTHA, we measured levels of RANKL, osteoprotegerin (OPG) and TRAP in whole paw tissue. We found that levels of both RANKL and TRAP increased in the arthritic paws on day 4 and 7, and the RANKL/OPG ratio increased after T_reg_ depletion (Fig. 3C). We also investigated the cellular players in the increased inflammatory response after T_reg_ depletion. Systemic neutrophilia and increased numbers of neutrophils in inflamed paws and dPLNs were observed (Fig. 4A,B). Increased numbers of macrophages in the inflamed paws and dPLNs were also observed (Fig. 4B and data not shown). Numbers of CD4^+^ T cells in the paw were increased compared to controls on day 7 but not day 4 (Fig. 4B), but there was no increase in CD4^+^ T cells in the dPLN (Fig. 4C). However, CD4^+^ T cells in the dPLN were found to be more activated (Fig. 4D). Taken together, these data show that depletion of T_regs_ increases systemic inflammation, increases the production of autoantibodies, increases bone resorption, increases the numbers of neutrophils and macrophages in the inflamed paw and its draining lymph node, and enhances T-cell activation.

### Concomitant depletion of T_regs_ and blockade of IL-10 signalling increased paw and ankle swelling to an even larger degree than T_reg_ depletion or IL-10 blockade alone

We found that concomitant depletion of T_regs_ and blockade of IL-10 signalling increased paw and ankle swelling to an even larger degree than T_reg_ depletion or IL-10 blockade alone (Fig. S3A-C). Active bone remodelling on day 10 post-arthritis induction, measured through serum CTX-I, was only detectable in mice with both depleted T_regs_ and blocked IL-10R signalling (Fig. S3D).

### Depletion of T_regs_ increases bacterial diversity transiently without affecting the overall major taxonomic composition of fecal microbiota

**Fig. 5.**
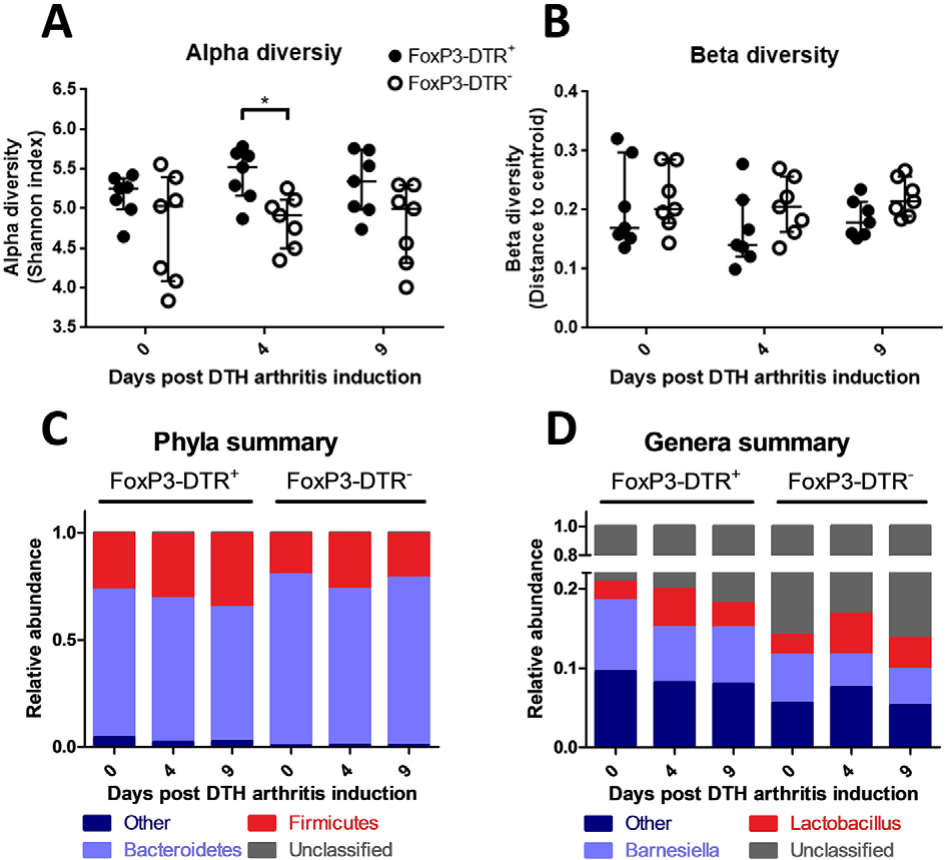
**T_reg_ depletion in DTHA has limited effect on the overall composition of the microbiota in the distal colon.** Fecal microbiota was analysed for bacterial 16S rRNA composition. (A) Alpha diversity based on Shannon index of unfiltered data for fecal samples. Median alpha diversity with upper and lower quantiles shown, *n*=7. *P≤0.05. (B) Beta diversity based on Whittaker's species turnover within each group. Median beta diversity with upper and lower quantiles indicated, *n*=7. (C) Taxa summary plot at phylum level showing changes in the microbiota composition of the fecal samples. Mean relative abundance shown, *n*=7. (D) Taxa summary plot at phylum level showing changes in the microbiota composition of the fecal samples. Mean relative abundance shown, *n*=7. Except for beta diversity, Wilcoxon rank-sum for tests between groups and Wilcoxon signed-rank for tests within groups. This experiment was carried out once.

Fecal samples from the distal colon were collected before DTHA induction on day 0, and on day 4 and 9 following DTHA induction with or without concurrent depletion of T_regs_. The composition of the microbiota was investigated by sequencing of the V4 hypervariable region of the 16S rRNA gene. Principal coordinate analysis (PCoA) at the level of operational taxonomic units (OTUs) revealed no significant changes in overall clustering following DTHA induction (Fig. S4). However, we found that the bacterial composition of the T_reg_-depleted mice was more diverse on day 4 compared to the non-depleted mice (Fig. 5A), with a tendency towards an increase in alpha diversity for the T_reg_-depleted mice from day 0 to 4 (*P*=0.141), whereas beta diversity did not differ significantly (Fig. 5B). Interestingly, alpha diversity correlated inversely with ankle swelling for the T_reg_-depleted group at day 4 (Spearman's ρ=−0.964; *P*<0.01; *n*=7). Analysis at the phylum level revealed no significant differences between the two groups, or between days within each group (Fig. 5C). We detected no significant changes in the relative abundance of genera (Fig. 5D) between groups, or between days within each group, although the abundance of *Lactobacillus* tended to be higher on day 4 compared to day 0 and 9 (*P*=0.084 and 0.12 for FoxP3-DTR^+^ and *P*=0.229 and 0.305 for FoxP3-DTR^−^, respectively). Correlations between taxa abundance and ankle swelling, paw swelling, TRAP, and CTX-I, respectively, did not show high correlation coefficients or patterns of correlations (data not shown). Taken together, these data show that DTHA induction alone or in conjunction with the depletion of T_regs_ induce only minor and transient compositional changes in the fecal bacterial microbiota. Thus, contrasting with results obtained by analysis of RA patients (Zhang et al., 2015), no significant arthritis-associated changes in the gut microbiota were detected in the mouse models.

### Depletion of T_regs_ leads to increased production of inflammatory mediators locally in the arthritic paws

**Fig. 6.**
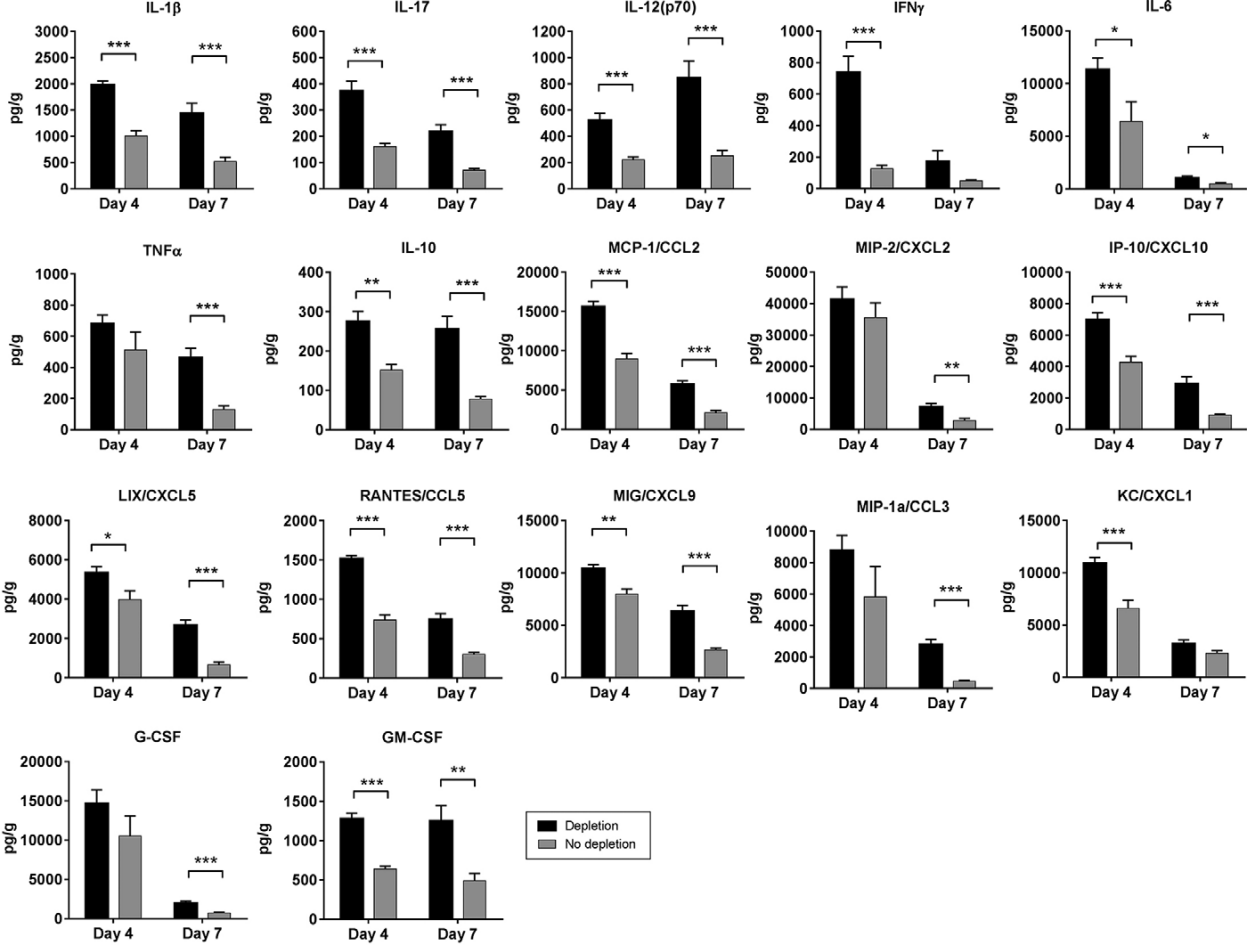
**Depletion of T_regs_ augments production of inflammatory mediators locally in the affected paws.** Whole paw homogenate supernatants were analysed for protein levels (pg/g tissue) of a range of inflammatory markers using multiplex analysis. Mean±s.e.m. shown, *n*=10. Student's *t*-test, *P≤0.05; **P≤0.01; ***P≤0.001. *P*-values were corrected for mass significance using the False Discovery Rate method. The raw and corrected *P*-values are shown in Table S1. MCP, monocyte chemoattractant protein; CCL, CC chemokine ligand; MIP, macrophage inflammatory protein; CXCL, CXC chemokine ligand; IP, interferon gamma-induced protein; MIG, monokine induced by gamma interferon; RANTES, regulated on activation, normal T cell expressed and secreted; G-CSF, granulocyte colony-stimulating factor; GM-CSF, granulocyte-macrophage colony-stimulating factor. Results presented in this figure are representative of two independent experiments.

To further elucidate the mechanisms of increased inflammation following T_reg_ depletion in DTHA, we measured levels of a range of cytokines and chemokines in whole paw tissue. The cytokines IL1β, IL-17, IL-12(p70), IL-6 and IL-10 were all increased in paw tissue on both day 4 and 7 compared with controls (Fig. 6). IFNγ was increased only on day 4 and TNFα only on day 7 (Fig. 6). The chemokines CCL2 (MCP-1), CXCL10 (IP-10), CXCL5 (LIX), CCL5 (RANTES) and CXCL9 (MIG) were all increased in paw tissue on both day 4 and 7 compared with controls (Fig. 6). CXCL2 (MIP-2), CCL3 (MIP-1α) and CXCL1 (KC) were increased only on day 7 and tended to increase on day 4 (Fig. 6). Levels of G-CSF were increased in paw tissue on day 7 and tended to increase on day 4 (Fig. 6). Granulocyte-macrophage colony-stimulating factor (GM-CSF) was increased in paw tissue both on day 4 and 7 after DTHA induction (Fig. 6). Taken together, these data show a profile of inflammatory mediators that both favours and is a result of Th17 T-cell polarisation, T-cell infiltration and activation, and neutrophil and macrophage infiltration and activation.

### Treatment with anti-IL-17 mAb rescues mice from exacerbation of disease caused by depletion of T_regs_

**Fig. 7.**
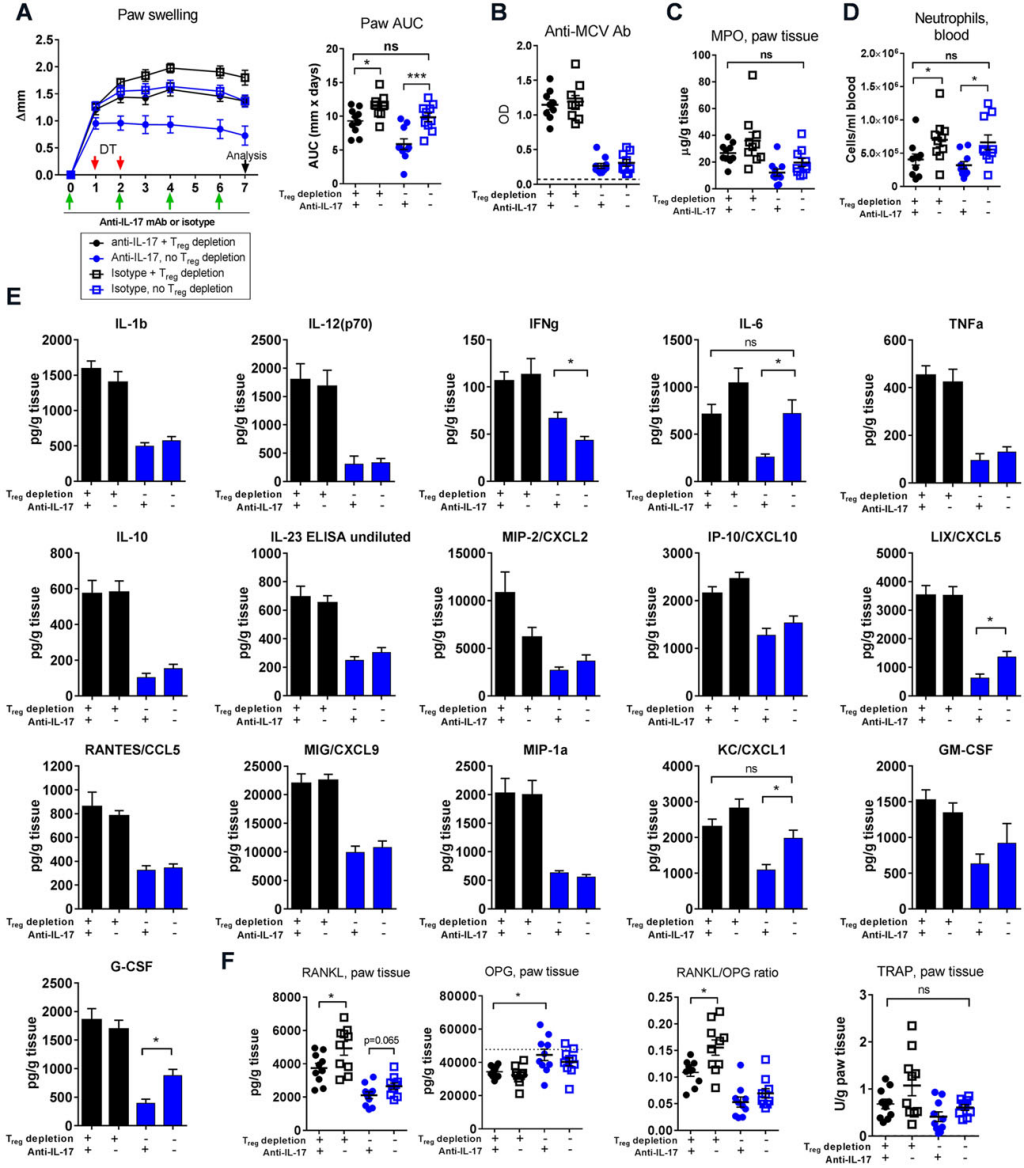
**Treatment with anti-IL-17 rescues mice from exacerbated disease caused by T_reg_ depletion.** (A) Anti-IL-17 treatment was initiated at the time of arthritis induction 1 day prior to T_reg_ depletion and given every 48 h until experiment termination on day 7. Left graph: paw swelling day 0-7. Right graph: area under curve (AUC) of paw swelling day 0-7. ns, not significant, *P≤0.05, ***P≤0.001. (B) Levels of anti-mutated-citrullinated-vimentin (MCV) antibodies in serum measured by ELISA as optical density (OD). Dotted line represents levels in *n*=5 naïve mice. (C) Levels of myeloperoxidase (MPO) in paw tissue measured by ELISA of whole paw homogenate supernatants. (D) Neutrophils/ml blood measured by flow cytometry. Neutrophils were defined as live CD45^+^TCRβ^−^CD11b^+^Ly6G^+^ cells. ns, not significant, *P≤0.05. (E) Analysis of inflammatory mediators in whole paw homogenate supernatants from T_reg_-depleted mice (blue bars) and non-depleted mice (black bars) treated with anti-IL-17 mAb or isotype control, using multiplex analysis [IL-1β, TNFα, IFNγ, IL-6, IL-10, IL-12(p70), MIP-2 (CXCL2), IP-10 (CXCL10), LIX (CXCL5), RANTES (CCL5), MIG (CXCL9), MIP-1α (CCL3), KC (CXCL1), G-CSF and GM-CSF] and ELISA (IL-23). Mean±s.e.m. shown, *n*=10. ns, not significant, *P≤0.05, Student's *t*-test. *P*-values were corrected for mass significance using the False Discovery Rate method. The raw and corrected *P*-values are shown in Table S1. This experiment was carried out once. (F) Levels of RANKL, OPG and TRAP were measured in whole paw homogenate supernatants from T_reg_-depleted mice (blue) and non-depleted mice (black) treated with anti-IL-17 mAb or isotype control, using ELISA. Mean±s.e.m. shown, *n*=10. ns, not significant, *P≤0.05. Dotted line represents mean OPG level in *n*=2 naïve mice. CCL, CC chemokine ligand; MIP, macrophage inflammatory protein; CXCL, CXC chemokine ligand; IP, interferon gamma-induced protein; G-CSF, granulocyte colony-stimulating factor; GM-CSF, granulocyte-macrophage colony-stimulating factor; MIG, monokine induced by gamma interferon; OPG, osteoprotegerin; RANKL, receptor-activator of nuclear factor κB ligand; RANTES, regulated on activation, normal T cell expressed and secreted; TRAP, tartrate-resistant acid phosphatase.

Based on our findings so far, we hypothesised that IL-17 could play a part in the increased disease activity observed following T_reg_ depletion. To test this, we set up a study where T_reg_-depleted mice were also treated with anti-IL-17 mAb. We found that anti-IL-17 treatment reduced paw swelling in mice both with and without intact T_reg_ compartments (Fig. 7A). We also found that anti-IL-17 treatment of T_reg_-depleted mice reduced paw swelling to levels seen in isotype-treated, non-T_reg_-depleted mice, thus rescuing them from the exacerbated disease caused by the depletion of T_regs_ (Fig. 7A). Starting anti-IL-17 treatment at immunisation (Fig. S5) did not reduce paw swelling to a greater degree than anti-IL-17 treatment begun at the time of arthritis induction (Fig. 7A). Anti-IL-17 treatment did not have an effect on the production of anti-MCV (Fig. 7B). There was a tendency towards reduced myeloperoxidase (MPO) levels in anti-IL-17-treated mice both with and without intact T_reg_ compartments, compared with isotype-control-treated animals (Fig. 7C). In addition, neutrophil numbers in circulation were reduced after IL-17 blockade in mice both with and without an intact T_reg_ compartment (Fig. 7D). We found that blockade of IL-17 reduced levels of IL-6, CXCL1, CXCL5 and G-CSF in arthritic paws of mice with intact T_reg_ compartments and tended to reduce IL-6 and CXCL1 in mice depleted of T_regs_ (Fig. 7E). IL-17 blockade reduced the levels of IL-6 and CXCL1 in mice that received T_reg_ depletion+anti-IL-17 to levels seen in non-T_reg_-depleted+isotype-treated mice (Fig. 7E). Importantly, anti-IL-17 treatment reduced levels of RANKL in paw tissue in mice both with and without intact T_reg_ compartments (Fig. 7F). In line with this, there was a tendency towards a reduction of TRAP levels after IL-17 blockade. T_reg_ depletion reduced the RANKL/OPG ratio and IL-17 blockade increased the RANKL/OPG ratio, indicating that T_reg_ depletion favours a more resorptive state, whereas IL-17 blockade reverses the effect of T_reg_ depletion on bone erosion (Fig. 7F). In summary, we find that IL-17 blockade rescues mice from the increased disease activity observed after T_reg_ depletion, and does so through a reduction in neutrophil numbers and local IL-6, CXCL1 and RANKL levels.

## DISCUSSION

In RA, dysregulation of the T_reg_ compartment has been reported, and T_reg_ numbers and levels of IL-17 expression correlate with disease severity (Noack and Miossec, 2014; Zaiss et al., 2010). Different approaches to tipping the T_eff_/T_reg_ balance towards T_reg_ dominance have been put forward as potential therapeutic strategies in RA and other inflammatory diseases; however, none have yet been fully successful (Miyara et al., 2014). Knowledge of the roles played by T_regs_ in experimental arthritis models is instrumental for the development of safe and effective therapeutic strategies involving T_regs_ in humans. In this study, we investigated the kinetics of T_regs_ in DTHA and characterised the exacerbated inflammatory response that resulted from selective depletion of T_regs_. To our knowledge, this is the first study to characterise the mechanisms of exacerbated disease in an arthritis model following depletion of T_regs_ using *Foxp3-DTR-eGFP* mice. Transient DT treatment, although sufficient to give the desired biological effect in many cases, can also leave a residual number of eGFP^+^ FoxP3^+^ cells, and these cells eventually repopulate the T_reg_ niche (Lahl et al., 2007; Lahl and Sparwasser, 2011). However, because we were interested in examining the effects of T_reg_ depletion on inflammation in the early stages of DTHA, our assessment was that this mouse was suitable for our purpose, despite its limitations.

T_reg_ numbers in DTHA expanded rapidly and these cells were found to be highly activated and proliferating in lymphoid and paw tissue. This indicates that T_regs_ are important in the early stages of DTHA. However, the fact that we observed severe inflammation in the affected paw despite the expansion of T_regs_ could be the result of a dysregulation of the Th17/T_reg_ balance, because increased Th17 activity is also seen in the early stages of DTHA (Atkinson et al., 2012). In support of this, we also show in the present study that neutralisation of IL-17 reduced severity of disease and could prevent the detrimental effects of T_reg_ depletion in the model.

Selective T_reg_ depletion increased arthritis severity, and the observed correlation between paw inflammation and new bone formation indicated that new bone formation could be linked to inflammation in DTHA. In support of this, we have previously shown that suppression of inflammation in DTHA with anti-TNFα antibodies (Atkinson et al., 2012) or anti-C5aR antibodies (Atkinson et al., 2015) could reduce bone formation. Inflammation has also been linked to bone formation in the CIA, proteoglycan-induced arthritis (PGIA) and SKG inflammatory arthritis models (Denninger et al., 2015; Schett et al., 2009; Haynes et al., 2012; Keller et al., 2012), and in ankylosing spondylitis (Maksymowych et al., 2013; Wanders et al., 2005). However, T_reg_ depletion also led to increased bone resorption in DTHA in the present study. The presence of these two opposing phenotypes can seem counterintuitive, but the bone formation and bone erosion take place at different areas of the paw (Atkinson et al., 2012), and simultaneous bone erosion and bone formation has also been observed in the CIA model in a recent study (Denninger et al., 2015). The net effect, however, is a negative association between inflammation and bone mass, as previously described (Gilbert et al., 2013; Proulx et al., 2007; Walsh et al., 2005).

Depletion of T_regs_ led to increased CD4^+^ T-cell activation and to increased recruitment of CD4^+^ T cells to the site of inflammation. This shows that the control of T_eff_ activation in the dPLN and T_eff_ numbers in the paw is one of the mechanisms of T_reg_-mediated suppression of DTHA. We also observed increased levels of anti-MCV, demonstrating an augmented immune response to a citrullinated self-antigen. Anti-MCV antibodies and other anti-citrullinated protein antibodies (ACPAs) are used as prognostic biomarkers of disease and joint erosion in RA, and ACPA levels correlate with serum CTX-I in RA patients (Gudmann et al., 2015; Jilani and Mackworth-Young, 2015). The fact that we observe a break of self-tolerance to MCV in DTHA and that anti-MCV levels increase with increased disease severity adds to the strengths of DTHA as a model of RA. However, the current study did not address which, if any, role anti-MCV plays in the pathogenesis of DTHA.

Previous studies have shown that T_regs_ are able to limit the accumulation of Th17 cells at inflammatory sites and draining lymph nodes (Lohr et al., 2006). Increased IL-17 production was observed in the T_reg_-depleted mice in the present study. This is supported by the observed increase in production of IL-6, IL-23 and IL-1β, which are cytokines that favour Th17 polarisation (Lubberts et al., 2005; Sonderegger et al., 2008; Sutton et al., 2006). IL-17 is an important cytokine in experimental arthritis when T cells play a part in the pathogenesis (Atkinson et al., 2012; Lubberts et al., 2005). Blockade of IL-17 has previously been shown to ameliorate experimental arthritis and is also a promising therapeutic avenue in RA (Lubberts et al., 2001, 2004; Pöllinger et al., 2011; van den Berg and McInnes, 2013). We found that treatment with anti-IL-17 could attenuate disease in DTHA, and the results indicate that IL-17 is more important for DTHA pathogenesis after the antigen challenge. The increased IL-17 production after T_reg_ depletion could also contribute to the increased bone erosion observed, and results from other studies support this. IL-17 induces osteoclastogenesis from human monocytes in the absence of RANKL (Yago et al., 2009) and upregulates RANKL expression (Gizinski and Fox, 2014), and blocking IL-17 prevents joint destruction in CIA through an increased RANKL/OPG ratio (Lubberts et al., 2004). Our results thus suggest that the increase in bone erosion could be driven by an increase in the RANKL/OPG ratio driven by increased IL-17 activity.

We observed increased IL-10 production in arthritic paws after T_reg_ depletion, which suggests that the contribution of IL-10 by T_regs_ to immunoregulation in DTHA is negligible. In a study in which T_regs_ were depleted using anti-CD25 mAb, we found that concomitant blockade of IL-10 signalling increased paw and ankle swelling to an even larger degree than T_reg_ depletion or IL-10 blockade alone. In the absence of T_regs_, regulatory B cells and their release of IL-10 could represent a compensatory regulatory pathway. B-cell-derived IL-10 has been shown to regulate inflammation in other mouse models (Fillatreau et al., 2002; Mauri et al., 2003; Mizoguchi et al., 1997). In experimental autoimmune encephalitis (EAE), components from *Mycobacterium tuberculosis* in the complete Freund's adjuvant (CFA) that is used to induce disease provide Toll-like receptor (TLR) agonists that can trigger regulatory functions of B cells that are required for the resolution of EAE (Lampropoulou et al., 2008).

Because B cells are not required for the induction of DTHA (Tanaka et al., 2007) and because CFA containing *M.* *tuberculosis* is also used for induction, it could be speculated that B cells indeed play an immunoregulatory role in DTHA and that depletion of B cells on top of T_reg_ depletion would further exacerbate DTHA.

We also observed an increased number of neutrophils in paws, dPLNs and blood as well as increased production of granulopoietic factors and neutrophil chemoattractants after T_reg_ depletion. This could in part be driven by the increased IL-17 production, because IL-17 is known to stimulate neutrophil recruitment through induction of G-CSF, GM-CSF and chemoattractants, including CXCL1 (Hamilton, 2008; Kolls and Lindén, 2004; Park et al., 2005; Schwarzenberger et al., 2000). Increased levels of CXCL1 and CXCL2 at the site of inflammation have also previously been shown to result from T_reg_ depletion in mice (Richards et al., 2010). Indeed, blockade of IL-17 reduced neutrophil numbers in T_reg_-depleted mice and reduced levels of CXCL1. IL-6 production can be induced by IL-17 (Kolls and Lindén, 2004) and, in line with this, we observed a major decrease of IL-6 production following IL-17 blockade. IL-6 plays an important role in neutrophil trafficking (Fielding et al., 2008; Lally et al., 2005), so the observed decrease in blood neutrophil numbers after IL-17 blockade could be a result of the decreased IL-6 levels. Taken together, these data support a regulatory role for IL-17 in neutrophil recruitment through IL-6 and CXCL1.

Recent findings have suggested an association between the gut microbiota and RA (Scher et al., 2013; Zhang et al., 2015), and interactions between the gut microbiota and immune function, including T_regs_, neutrophils and IL-17, are well established (Maynard et al., 2012). However, in our model, depletion of T_regs_ did not significantly change the bacterial composition, indicating that immune-driven changes in the gut microbiota are unlikely to play an important role in disease progression in our model. We observed a tendency towards higher alpha diversity in T_reg_-depleted mice and that alpha diversity correlated inversely with ankle size at day 4. Interestingly, we observed a tendency towards an increased abundance of *Lactobacillus* on day 4, which also correlated with maximal ankle swelling. *Lactobacillus* has been shown to be over-represented in RA patients compared to healthy subjects (Liu et al., 2013; Zhang et al., 2015), and the abundance of *Lactobacillus* was also shown to correlate with disease activity in RA patients. Furthermore, mono-colonisation with *Lactobacillus bifidus* was shown to be sufficient for inducing joint inflammation in a mouse model of spontaneous arthritis via TLR2- and TLR4-dependent signalling affecting the balance between T_reg_, Th17 and Th1 in the gut (Abdollahi-Roodsaz et al., 2008). When comparing the microbial changes observed in laboratory animals to humans, it is important to consider the marked environmental differences. Moreover, in DTHA, severe disease is observed after just a few days, whereas RA develops over several years. Although the composition of the microbiota can change within days, the short duration of the model might not be sufficient to markedly affect the composition of the gut microbiota in a manner that reflects the situation in human disease pathogenesis. Nevertheless, the findings suggest a link between *Lactobacillus* and joint inflammation both in rodent models and RA patients.

### Conclusion

Taken together, our findings show that T_regs_ are important for the containment of inflammation in DTHA, and that T_reg_ depletion in DTHA acts, at least partly, through increasing IL-17 and RANKL activity and through increasing numbers of neutrophils in blood, dPLNs and paws. We show that IL-17 drives an increase in inflammation in the absence of T_regs_: administration of anti-IL-17 mAb just prior to T_reg_ depletion rescues mice from the exacerbation of disease associated with T_reg_ depletion through a reduction in IL-6 and CXCL1 production, reduced neutrophil numbers and a reduction in the RANKL/OPG ratio. Thus, an imbalance in the T_reg_ and Th17 relationship could be a driver of inflammation in DTHA. To our knowledge, this is the first study using the *Foxp3-DTR-eGFP* mouse on a C57BL/6 (B6) background for T_reg_ depletion in an arthritis model, and we here demonstrate the usefulness of the approach and build upon the already existing body of work in this new model. Many genetically modified mice are bred on a B6 background, and can thus be combined with the *Foxp3-DTR-eGFP* C57BL/6 mouse, making possible the study of T_reg_ depletion in concert with the absence/overexpression of proteins relevant for disease pathways. However, the limitations of the *Foxp3-DTR-eGFP* mouse generated by BAC transgenesis must be kept in mind when choosing this model system. Given the relevance of T_regs_ in RA and the possibility of developing T_reg_-mediated therapies for RA, using this approach for selective depletion of T_regs_ could yield important results to help understand the diverse roles of T_regs_ in RA and other inflammatory diseases.

## MATERIALS AND METHODS

### Mice

Female C57BL/6J mice were purchased from Taconic, Ry, Denmark. Female *Foxp3-DTR-eGFP* mice were generated as described in Lahl et al. (2007), and bred and maintained at Forschungseinrichtungen für Experimentelle Medizin, Charité Universitätsmedizin Berlin or purchased from Jackson Laboratories (Bar Harbor, ME) on a NOD background (generated as described in Feuerer et al., 2009) and backcrossed for at least eight generations onto the C57BL/6 background at Taconic, Ry, Denmark. *Foxp3-DTR-eGFP* mice express a fusion protein consisting of the diphtheria toxin (DT) receptor (DTR) and enhanced green fluorescent protein (eGFP) under the control of the *Foxp3* gene locus inserted by BAC transgenesis. This construct allows selective depletion of T_regs_ by DT injection. Animals were housed in a facility with a 12-h light/dark cycle and with free access to water and standard rodent chow (Altromin^®^). All animal experiments performed in Germany were in accordance with the guideline 2010/63/EU from the European Union and the European Convention for the protection of vertebrate animals used for experimental and other scientific purposes. Animal protocols were approved by the ethics committee and the Berlin state authorities (LAGeSo registration # G0331/08). All animal experiments performed in Denmark were conducted according to Danish legislation and have been approved by the Danish Animal Inspectorate and the Novo Nordisk ethical review board.

### Induction and assessment of DTHA

Mice were anaesthetised by isoflurane/O_2_/N_2_O and immunised intradermally (i.d.) with methylated bovine serum albumin (mBSA) (Sigma, St Louis, MO) emulsified in complete Freund's adjuvant (CFA) (Difco, Detroit, MI) at the tail base. Four days later mice were given 1000 µg (approx. 50 mg/kg body weight) anti-mouse type II collagen antibody (anti-CII) cocktail (Chondrex, Redmond, WA) containing the clones A2-10 (IgG2a), F10-21 (IgG2a), D8-6 (IgG2a), D1-2G (IgG2b) and D2-112 (IgG2b) intravenously in 200 µl phosphate-buffered saline (PBS). Seven days after immunisation the mice were challenged with 200 µg mBSA subcutaneously in 20 μl PBS in the right foot pad. The left foot pad was given 20 μl PBS only and served as control. Baseline paw and ankle measurements were made on the right paw on day 0 prior to mBSA challenge. Paw and ankle swelling was measured using a dial thickness gauge (Mitutoyo, Japan), and was calculated as right paw or ankle thickness minus baseline measurement.

### Depletion of T_regs_

Mice were injected intraperitoneally with 1 µg DT (Merck, Darmstadt, Germany) in 100 µl PBS 24 and 48 h after DTHA induction. Depletion of T_regs_ was confirmed by flow cytometry on whole blood sampled 24 h after the final DT dose (Fig. S1 shows a representative depletion check). Cells were gated on TCRβ, CD4 and CD25 and analysed for expression of eGFP. Antibodies used were anti-CD16/32 (BD Biosciences, NJ) for blocking of unspecific Fc-receptor binding, anti-TCRβ-PerCP-Cy5.5, clone H57-597 (eBioscience, San Diego, CA), anti-CD25-APC, clone PC61.5 (eBioscience, San Diego, CA), anti-CD4-PE-Cy7, clone RM4-5 (BD Biosciences, NJ) or anti-CD4-Qdot605 (Invitrogen, Carlsbad, CA).

### Depletion of T_regs_ and IL-10R blockade

For depletion of T_regs_, mice were dosed with 200 µg rat anti-mouse CD25 mAb (clone PC.61, BioXcell, West Lebanon, NH) or rat IgG1 anti-trinitrophenol (TNP) isotype control (Novo Nordisk A/S, Måløv, Denmark) 11 and 8 days prior to immunisation. For blockade of IL-10R, mice were dosed with 250 µg rat anti-mouse IL-10R mAb (clone 1B1.3A, BioXcell, West Lebanon, NH) or rat IgG1 anti-TNP isotype control (Novo Nordisk A/S, Måløv, Denmark) three times weekly from the time of immunisation. Depletion of T_regs_ was confirmed by flow cytometry on whole blood samples drawn 48 h after the second dose of anti-CD25. Mouse regulatory T cell staining kit (eBioscience, San Diego, CA) was used. Antibodies used were CD45-PerCP, CD4-APC and CD25-FITC all from BD Biosciences (NJ), and FoxP3-PE (eBioscience, San Diego, CA).

### Anti-IL-17 mAb treatment

Mice were treated with 400 µg rat anti-mouse IL-17 mAb (clone 17F3, BioXcell, West Lebanon, NH) or rat IgG1 isotype control (clone MOPC-21, BioXcell, West Lebanon, NH) in 200 µl PBS on the day of arthritis induction and with 200 µg rat anti-mouse IL-17 mAb or isotype control in PBS after 48 h and every 48 h until the experiment was terminated on day 7. For results presented in Fig. S4, mice were dosed with 200 µg rat anti-mouse IL-17 mAb (clone 17F3, BioXcell, West Lebanon, NH) or rat IgG1 isotype control (clone MOPC-21, BioXcell, West Lebanon, NH) in 200 µl PBS from the time of immunisation and every 48 h until study termination.

### Histopathology

Paws were processed and stained with hematoxylin and eosin (H&E), Safranin O and for tartrate-resistant acid phosphatase (TRAP) as previously described (Atkinson et al., 2012). TRAP stains osteoclasts red, and Safranin O stains cartilage red. The intensity of Safranin O staining is directly proportional to the proteoglycan content in cartilage. Pathological changes in the paws were assessed on H&E-, TRAP- and Safranin-O-stained sections. The extra-articular infiltration of inflammatory cells (assessed on a scale of 0-3) and arthritic changes were assessed separately. Arthritic changes were assessed on metatarsal and tarsal joints as synovitis, cartilage destruction and bone erosion, and scored separately on a 0-3 scale. For each of the three parameters of arthritic changes, an average between the two joint areas was calculated. In addition, new bone formation and extra-articular infiltration overall in the paw was scored on a 0-3 scale. New bone formation was defined as the presence of osteophytes, as described in Atkinson et al. (2012). The histology sum score was calculated by adding the five scores (extra-articular infiltration, synovitis, cartilage destruction, bone erosion and bone formation), whereas the extra-articular infiltration score is left out in the arthritis score. The person who performed the evaluation was blinded to the experimental setup.

### Immunohistochemistry and digitalised image analysis

Immunohistochemical (IHC) detection of macrophages and neutrophils in the paws was performed as previously described (Atkinson et al., 2015). The sections were all digitally scanned and studied using a NanoZoomer Digital Pathology Virtual Slide Viewer (Hamamatsu Photonic, Shizuoka, Japan). Automated digital image analyses of the infiltrating macrophages (F4/80^+^) and neutrophils (Ly6B.2^+^) in the paws were performed using the Visiopharm Integrator System (VIS; version 4.2.2.0, Visiopharm, Hoersholm, Denmark). On individual digital images of the arthritic paw a region-of-interest (ROI) was automatically defined of the entire paw. The bone marrow was outlined manually and excluded from analysis, as was hair follicles and artefacts. Next, an analysis was run inside the ROI to detect the brown DAB (3-3′-diamino-benzidine-tetrahydrochloride) staining of the target cells. The results are given as percentage of tissue area positive for F4/80 or Ly6B.2 of the entire paw area.

### Flow cytometry

Single-cell suspensions of blood, popliteal lymph nodes and paw infiltrate were prepared as previously described (Atkinson et al., 2012). All samples were subjected to Fc-blocking prior to antibody staining using anti-CD16/32 (BD Biosciences, NJ). Dead cells were excluded using Fixable Near-IR Vital dye (Invitrogen, Carlsbad, CA). Antibodies used were anti-CD45-V500 (clone 30-F11, BD Biosciences, NJ), anti-CD45-eFluor450 (clone 30-F11, eBioscience, San Diego, CA), anti-TCRβ-PerCP-Cy5.5 (clone H57-597, eBioscience, San Diego, CA), anti-TCRβ-Qdot655 (clone H57-597, Molecular Probes, Carlsbad, CA) anti-CD4-PE-Cy7 (clone RM4-5, BD Biosciences, NJ), anti-CD4-Qdot605 (clone RM4-5, Invitrogen, Carlsbad, CA), anti-CD19-FITC (clone 1D3 eBioscience, San Diego, CA), anti-CD62L-PE (clone MEL-14, eBioscience, San Diego, CA), anti-CD44-APC (clone IM7, eBioscience, San Diego, CA), anti-CD8a-Pacific Blue (clone 53-6.7, Biolegend, San Diego, CA), anti-F4/80-eFluor450 (clone BM8, eBioscience, San Diego, CA), anti-Ly6.G-PE (clone 1A8, BD Biosciences, NJ) and anti-CD11b-APC (clone M1/70, eBioscience, San Diego, CA). Samples were run on an LSRII or FACSCanto flow cytometer and data were analysed using FACSDiva software (BD Biosciences, NJ). All cells were gated on live/dead marker and CD45 before further analysis. Neutrophils were defined as CD11b^+^Ly6G^+^, macrophages as CD11b^+^F4/80^+^, T cells as TCRβ^+^, and CD4 and CD8 T cells as TCRβ^+^CD4^+^ and TCRβ^+^CD8^+^, respectively. Full gating strategies for the individual experiments are shown in Fig. S1.

### Multiplex analysis of inflammatory markers in paw homogenate

Hind paws were sampled at selected times after DTHA induction and each paw was placed in 1.25 ml of an ice-cold custom-made homogenization buffer containing a solution of 200 mM NaCl, 5 mM EDTA, 10 mM Tris, 10% glycerin, 1 mM phenylmethylsulfonyl fluoride (PMSF), 1 mg/ml leupeptin and 28 mg/ml aprotinin with a pH value of 7.4 (Ampliqon, Skovlunde, Denmark). The paws were homogenized by using a T25 Ultraturrax homogeniser (IKA, Staufen, Germany) followed by centrifugation at 10,000 ***g*** for 15 min. The supernatants were decanted and centrifuged once more at 10,000 ***g*** for 15 min. The final supernatants were analysed undiluted for levels of IL-1β, TNFα, IFNγ, IL-6, IL-10, IL-17, IL-12(p40), MIP-2 (CXCL2), IP-10 (CXCL10), LIX (CXCL5), RANTES (CCL5), MIG (CXCL9), MIP-1α (CCL3), KC (CXCL1), MCP-1 (CCL2), G-CSF and GM-CSF using bead-based Luminex^®^ xMAP^®^ technology with Milliplex kits from Millipore (Billerica, MA) according to the manufacturer's instructions. For statistical analysis, any values below the detection limit were set to the detection limit for the analyte in question and any values above the detection limit were set to the upper detection limit for the analyte in question.

### Enzyme-linked immunosorbent assays (ELISAs)

Levels of serum amyloid P component (SAP) were measured in serum using kits from Genway (San Diego, CA) according to the manufacturer's instructions. Levels of receptor-activator of nuclear factor kappa B (RANKL), osteoprotegerin (OPG) and IL-23 were measured in paw homogenate supernatants using kits from R&D Systems (Minneapolis, MN) according to the manufacturer's instructions. Levels of CTX-I and TRAP were measured in serum and paw homogenate supernatants using ELISA kits from Immunodiagnostic Systems (Boldon, UK) according to the manufacturer's instructions. Levels of MMP3, IL-6 and G-CSF were measured in serum using kits from R&D Systems (Minneapolis, MN) according to the manufacturer's instructions. Antibodies to mutated citrullinated vimentin (anti-MCV) were measured using kits from Orgentec Diagnostika GmbH (Mainz, Germany) with the modification that the detection antibody supplied in the kit was substituted with goat anti-mouse IgG-HRP (Invitrogen, Carlsbad, CA) diluted 1:3000 and the substrate solution supplied was substituted with substrate reagents from R&D Systems (Minneapolis, MN).

### mRNA deep sequencing

Arthritic hind paws were removed at the hairline and homogenised in Qiagen RLT buffer (Qiagen, Germantown, MD) with 1% 2-betamercaptoethanol (Sigma-Aldrich, St Louis, MO) and stored at −80°C. The Ambion Magmax prep protocol was used to extract total RNA. RNA quantity was assessed using the Nanodrop and RNA quality was assessed using the Agilent Bioanalyzer (Agilent Technologies, Santa Clara, CA). RNAseq libraries were prepared using the Illumina TruSeq Sample Prep Kit (Illumina, Inc., San Diego, CA). 120 ng of total RNA was used for input. Barcode adapters were added to samples in such a way as to allow pooling of samples in flow cell lanes in a randomized pattern relative to sample annotation and source. Samples were sequenced using an Illumina HiSeq 2000 (Illumina, Inc., San Diego, CA) at a multiplexing level sufficient to generation 10-million to 25-million reads per sample. Following generation of sequence, reads were aligned to the mouse genome version NCBI m37 using TopHat (http://tophat.cbcb.umd.edu/). Quality control of sequencing data was accomplished by using the ShortRead package in R (http://www.r-project.org/). Aligned reads were mapped to Ensembl transcript models and converted to reads per kilobase of transcript per million mapped reads (RPKM) values using Cufflinks (http://cufflinks.cbcb.umd.edu/).

### Bacterial 16s rDNA sequencing

Bacterial DNA from fecal samples from the distal colon was extracted using the NucleoSpin soil kit (Macherey-Nagel, Düren, Germany) following the manufacturer's protocol. Yield and integrity of the DNA were assessed by Nanodrop and agarose gel electrophoresis, respectively. 16S rDNA amplification and library generation were performed as previously described (Holm et al., 2015). PCR products were purified using Agencourt AMPure XP beads (Beckman Coulter, Brea, CA) and normalized to 16-21 ng/μl per sample. Subsequently, samples were pooled (5 μl of each sample) and quantified using a Qubit dsDNA HS Assay Kit and a Qubit 2.0 Fluorometer (Life Technologies, Carlsbad, CA). 14 pM library and 0.7 pM PhiX Control v3 (Illumina, San Diego, CA) were sequenced using an Illumina MiSeq V2 PE500 cartridge (500 cycles) on an Illumina MiSeq. Sequences were analysed using QIIME v1.7.0 with default settings including quality-based sequence trimming, removal of primers, assembly of paired-end sequences and chimera checking (Caporaso et al., 2010). *De novo* operational taxonomic unit (OTU)-picking was performed by UCLUST (Edgar, 2010) utilizing 97% sequence similarity. OTUs were assigned against the Greengenes database v11_2 (DeSantis et al., 2006) using the RDP-classifier (Wang et al., 2007) with an 80% confidence threshold. Analyses were performed in R v3.2.0 using the metagenomeSeq (Paulson et al., 2013), PhyloSeq (McMurdie and Holmes, 2013) and Vegan (Oksanen et al., 2011) packages. Data was filtered for low-abundant OTUs by removing OTUs present in fewer than three of the 42 samples and with a relative abundance across all samples ≤0.5%. Analyses on filtered data were performed with an average of 20,731±7655 (s.d.) sequences per sample compared to an initial 24,974±9382 (s.d.) sequences before filtering. Alpha diversity (Shannon index) was calculated on unfiltered data. Read counts were normalized using metagenomeSeq (Paulson et al., 2013), which utilizes a cumulative-sum scaling where raw counts are divided by the cumulative sum of counts up to a particular quantile. Principal coordinate analysis (PCoA) using Bray-Curtis dissimilarity indices, analysis of differential abundance of taxa and beta diversity analysis using Whittaker's species turnover were performed on filtered and normalized data.

### Statistics

Statistical analyses were conducted using GraphPad Prism software version 6.04. Non-parametric data or non-normal parametric data were analysed using the Mann–Whitney *U*-test, and parametric data were analysed using a two-sided unpaired Student's *t*-test or one-way ANOVA. For statistical analysis of the histology score data, a two-sided unpaired Student's *t*-test with Welch's correction was used. For bacterial 16S rDNA, statistical analyses were conducted using R differences in abundance of phyla, alpha diversity measures and beta diversity between and within the FoxP3-DTR^−^ and FoxP3-DTR^+^ groups over the course of the study using the Wilcoxon Rank-Sum and Wilcoxon Signed-Rank test, respectively, with Benjamini–Hochberg *P*-value adjustment. Adonis test of significance was performed using the calculated Bray–Curtis distance matrix, to test for overall differences between the FoxP3-DTR^−^ and FoxP3-DTR^+^ groups and within each group over the course of the study. Correlation analyses between taxonomic abundances, alpha diversity, serum markers, and paw and ankle size were performed in R using Spearman's rank correlation test with Benjamini–Hochberg *P*-value adjustment. Differences between groups and correlation coefficients were considered significant when *P*≤0.05 and levels of significance were assigned as *P≤0.05, **P≤0.01 and ***P≤0.001. In Figs 6 and 7E *P*-values were corrected for mass significance using the False Discovery Rate method. The raw and corrected *P*-values are shown in Table S1.
